# Treating Refractory Hypertension: Renal Denervation With High-Resolution 3D-Angiography

**DOI:** 10.5812/cardiovascmed.9700

**Published:** 2013-05-20

**Authors:** Eduardo Alegria-Barrero, Rodrigo Teijeiro, Miguel Casares, Mercedes Vega, Marco A Blazquez, Ramon Martos, Carlos De Diego, Raúl Moreno, Miguel A San Martin

**Affiliations:** 1 Department of Cardiology and Interventional Cardiology, Torrejon University Hospital, Madrid, Spain; 2 Department of Radiology and Interventional Radiology, Torrejon University Hospital, Madrid, Spain; 3 Department of Cardiology and Interventional Cardiology, La Paz University Hospital, Madrid, Spain

**Keywords:** Hypertension, Cardiovascular Disease, Denervation, Angiography

## Abstract

A 53-year-old male was referred to our Department for refractory primary hypertension. Despite high doses of 6 anti-hypertensive drugs, ambulatory monitoring of blood pressure (BP) revealed a mean BP of 160/90 mmHg. Under local anaesthesia, renal denervation with radiofrequency was performed supported by high-resolution 3D angiography, which helped confirm the position of the applications in a spiroid fashion.

## 1. Introduction

High blood pressure is an independent cardiovascular risk factor with high prevalence (1). Precisely, hypertension can lead to ischemic heart disease (myocardial infarction), heart failure, stroke, dementia and chronic kidney disease (2). Despite physical exercise, dietary recommendations and pharmacological therapy, up to 50% of hypertensive patients do not achieve adequate blood pressure control (2). Clinical and experimental studies have shown a sympathetic activation with positive feedback as a physiopathological mechanism leading to hypertension and contributing to maintaining high blood pressure (3-5). Endovascular treatment with low dose radiofrequency in the renal arteries (renal denervation) has shown to be effective reducing sympathetic activation of efferent nerves, involved in hypertensive responses (6-9). Moreover, this technique has been shown to have prolongued predictable effects and low incidence of complications (mainly vascular access-related) (6). Blood pressure reduction has been effective in more than 85% of the subjects treated with renal denervation, adequately selected (systolic blood pressure > 160 mmHg despite > 4 antihipertensive drugs including diuretics), obtaining a reduction > 10 mmHg in systolic blood pressure (6, 7). Imaging techniques are continuously improving to help interventionalists conduct this novel technique (6). We discuss the contribution of different imaging modalities to the success of the procedure.

## 2. Case Report

**Figure 1.**
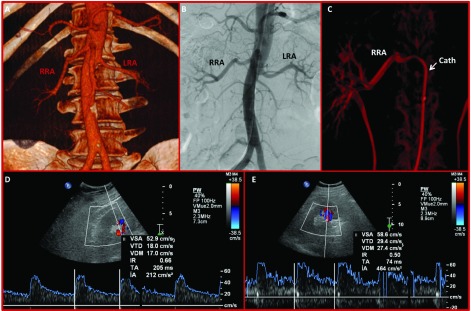
Multi-Modality Imaging For Renal Denervation A) 3D-angio-computerized tomography (CT) showing anatomic characteristics of the right and left renal arteries (RRA, LRA, respectively); B) Angiographic appearance of the renal arteries after contrast injection; C) 3D-angiography reconstruction of the RRA showing a curvature of the mid-RRA; D) RRA and E. LRA Doppler velocity showing normal waves

**Figure 2.**
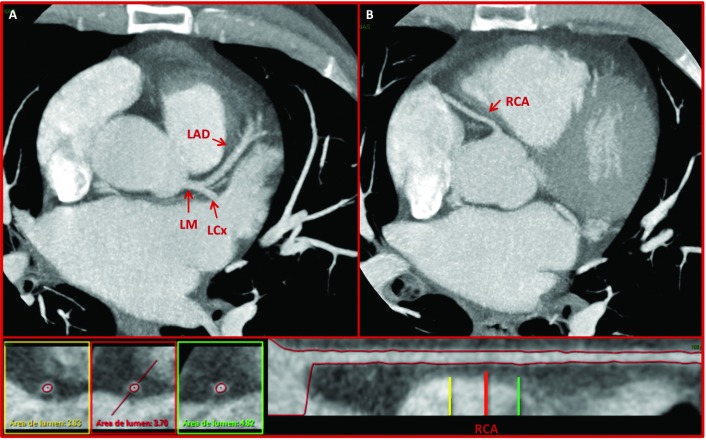
64-MDCT Coronary Angiography of the Patient Showing Normal Coronary Arteries

A 53-year-old Caucasian male was referred to our Department for refractory primary hypertension. Despite high doses of six anti-hypertensive drugs (Enalapril, Doxazosin, Hidroclorotiazide, Spironolactone, Amlodipine, Atenolol), ambulatory monitoring of blood pressure (BP) revealed a mean BP of 160/90 mmHg. Exhaustive blood tests, CT scan and renal arteries ultrasound suggested primary hypertension as the most probable diagnosis (Figure 1). Coronary CT scan ruled out significant coronary heart disease (Figure 2). Under local anaesthesia and after informed consent, renal denervation with radiofrequency was performed supported by high-resolution 3D angiography (Figure 1C), which helped confirm the position of the applications with Ardian´s Simplicity Catheter (Minneapolis, MN, USA) in an spiroid fashion and negotiate properly the curve of the right renal artery. 3D angiography was obtained after continuous contrast injection and 360º angiographic acquisition using a single-plane Phillips Allura Xper FD20, with XperSwing technology. Six weeks after the procedure, the patient has recovered without complications and systolic blood pressure has dropped 20 mmHg, measured by 24-hour ambulatory recording of blood pressure.

## 3. Discussion

Renal denervation has been proposed as an effective treatment for refractory primary hypertension for patients with inadequate blood control despite high doses of numerous drugs (9). Simplicity Trial (6, 7) was a multicentric randomized Trial that revealed a consistent, predictable and durable reduction of blood pressure after radiofrequency ablation of both renal arteries, following a spiroid fashion aiming to reduce sympathetic hyper activation of the efferent nervous terminations located at the media layer of the artery. After renal denervation, patients experience a progressive reduction of blood pressure, up to SBP reductions of 30-40 mmHg after 2 years (10), leading to a better blood pressure control, reducing pharmacological therapy. In addition, cost-effectiveness studies have demonstrated that this percutaneous treatment is cost-effective in the long-term. Imaging modalities are essential for interventionists aiming to deliver radiofrequency in a spiroid fashion. CT renal artery scan helps physicians determine the uptake of the arteries from the aorta the anatomy of both arteries. In the reported case, 3D angiographic reconstruction (Figure 1C) helped understand right renal artery anatomy and was used as guidance in order to negotiate the curve of the artery, enabling us to deliver radiofrequency satisfactory.
